# Aging-associated formaldehyde-induced norepinephrine deficiency contributes to age-related memory decline

**DOI:** 10.1111/acel.12345

**Published:** 2015-04-11

**Authors:** Yufei Mei, Chun Jiang, You Wan, Jihui Lv, Jianping Jia, Xiaomin Wang, Xu Yang, Zhiqian Tong

**Affiliations:** 1 Alzheimer’s disease Center, Beijing Institute for Brain Disorders, Capital Medical UniversityBeijing, 100069, China; 2 Section of Environmental Biomedicine, Hubei Key Laboratory of Genetic Regulation and Integrative Biology, College of Life Sciences, Central China Normal UniversityWuhan, 430079, China; 3 Neuroscience Research Institute & Department of Neurobiology, School of Basic Medical Sciences, Peking UniversityBeijing, 100191, China; 4 Beijing Geriatric HospitalBeijing, 100049, China

**Keywords:** formaldehyde, long-term potentiation, norepinephrine, senescence-accelerated prone 8, spatial memory, spontaneous discharge

## Abstract

A norepinephrine (NE) deficiency has been observed in aged rats and in patients with Alzheimer’s disease and is thought to cause cognitive disorder. Which endogenous factor induces NE depletion, however, is largely unknown. In this study, we investigated the effects of aging-associated formaldehyde (FA) on the inactivation of NE *in vitro* and *in vivo*, and on memory behaviors in rodents. The results showed that age-related DNA demethylation led to hippocampal FA accumulation, and when this occurred, the hippocampal NE content was reduced in healthy male rats of different ages. Furthermore, biochemical analysis revealed that FA rapidly inactivated NE *in vitro* and that an intrahippocampal injection of FA markedly reduced hippocampal NE levels in healthy adult rats. Unexpectedly, an injection of FA (at a pathological level) or 6-hydroxydopamine (6-OHDA, a NE depletor) can mimic age-related NE deficiency, long-term potentiation (LTP) impairments, and spatial memory deficits in healthy adult rats. Conversely, an injection of NE reversed age-related deficits in both LTP and memory in aged rats. In agreement with the above results, the senescence-accelerated prone 8 (SAMP8) mice also exhibited a severe deficit in LTP and memory associated with a more severe NE deficiency and FA accumulation, when compared with the age-matched, senescence-resistant 1 (SAMR1) mice. Injection of resveratrol (a natural FA scavenger) or NE into SAMP8 mice reversed FA accumulation and NE deficiency and restored the magnitude of LTP and memory. Collectively, these findings suggest that accumulated FA is a critical endogenous factor for aging-associated NE depletion and cognitive decline.

## Introduction

Hippocampal norepinephrine (NE) has, in recent decades, become well known as a neurotransmitter that is required for learning and memory (Gelinas & Nguyen, 2007). This is based on the fact that an injection of NE or an antagonist of NE receptor can enhance or block hippocampal long-term potentiation (LTP) and memory formation (Gray & Johnston, 1987; Izumi & Zorumski, 1999). Accumulating evidence indicates that memory decline associated with NE deficiency has been found in aged animals (such as mice and rats) and in Alzheimer’s disease (AD) TgCRND8 transgenic mice (Luine *et al*., 1990; Francis *et al*., 2012). Clinical investigations also show irregularities in NE content in the blood and cerebrospinal fluid (CSF) of older and patients with AD (Peskind *et al*., 1995; Szot *et al*., 2000), and a marked NE deficiency or norepinephrinergic neuron loss in the autopsied hippocampi and loci coerulei (LC) of AD patients (Chan-Palay & Asan, 1989). Furthermore, depletion of hippocampal NE by injection of 6-hydroxydopamine (6-OHDA, a NE depletor) or N-(2-chloroethyl)-N-ethyl-2-bromobenzylamine (DSP-4, a selective norepinephrinergic neurotoxin) has been found to induce deficits in LTP and memory in healthy adult rats (Stanton & Sarvey, 1985; Lapiz *et al*., 2001). All these data suggest that age-related NE deficiency contributes to cognitive disorder during aging and AD processes. However, which endogenous factor induces NE deficiency still needs to be elucidated.

An accumulation of endogenous formaldehyde (FA) has been extensively found in the hippocampi of aged mice and rats, the senescence-accelerated prone 8 (SAMP8) mice, APP and APP/PSI transgenic mice, and patients with AD (Tong *et al*., 2013b). Inhibiting FA degrading enzymes or injecting excess FA directly causes deficits in LTP and memory in healthy adult rat**s** (Tong *et al*., 2013a,b). Scavenging FA by injecting resveratrol (Res, a natural FA scavenger (Tyihak *et al*., 1998; Tong *et al*., 2011)), has been found to reduce hippocampal FA levels and reverse age-related memory deficits in aged rats and APP-transgenic mice (Karuppagounder *et al*., 2009). The above findings indicate that while the concentrations of these two endogenous factors (the accumulation of FA and an NE deficiency) change in the opposite direction, both of them have a common destructive effect: the impairment of memory during aging. However, the relationship between accumulated FA and NE deficiency is unclear. In this study, we hypothesize that accumulated FA induces hippocampal NE deficiency by inactivating NE, which in turn leads to memory decline during the aging process. We found that aging is associated with both FA accumulation and NE deficiency in the hippocampus. FA inactivates NE *in vitro* and *in vivo*. Scavenging FA from or supplying NE to the hippocampus can reduce memory deficits in aged rats. These findings suggest that accumulated FA results in a hippocampal NE deficiency and memory decline during aging.

## Results

### Aging is associated with DNA demethylation and FA accumulation in rats

**Fig 1:**
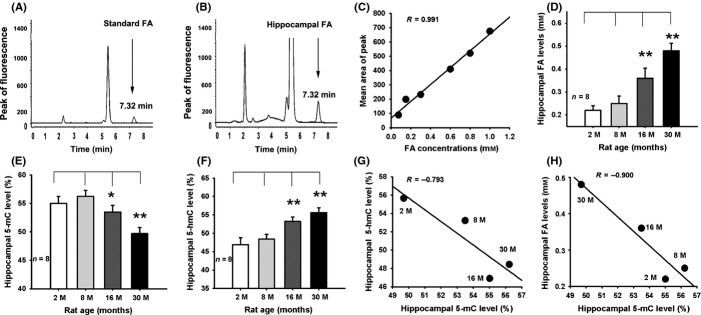
Aging leads to hippocampal DNA demethylation and FA accumulation. (A, B) The peak of fluorescence of standard FA and hippocampal FA. (C) The standard curve of FA (*n* = 6). (D) Hippocampal FA levels were elevated in aged rats (*n* = 8). (E, F) A decrease in 5-mC but an increase in 5-hmC concentrations in the hippocampi of rats of different ages (*n* = 8). (G) Negative correlation between 5-mC and 5-hmC concentration in hippocampus. (H) Negative correlation between 5-mC and FA level in the hippocampus. *P <* *0.05; **P <* *0.01.

To investigate whether age-related DNA demethylation leads to FA accumulation, we measured the changes in concentrations of FA, 5-methylcytosine (5-mC), and 5-hydroxymethylcytosine (5-hmC) in the hippocampi of rats at 2, 8, 16, and 30 months of age. This was based on previous studies that suggest that aging is associated with DNA demethylation (i.e., a decrease in 5-mC level) (Igor *et al*., 2010; Oliveira *et al*., 2012) and that DNA demethylation results in FA generation via ten-eleven translocation enzymes (TeTs) (Wu & Zhang, 2010; Guo *et al*., 2011) and demethylases (Patra *et al*., 2008) (Fig. S1A,B). Using high-performance liquid chromatography with a fluorescence detector (Fluo-HPLC) (Fig.1A–C), we found that there was a marked elevation in hippocampal FA concentrations in rats aged from 2 to 30 months (Fig.1D). Moreover, DNA methylation kit analysis showed that the aged rats at 30 months had a lower concentration of 5-mC, but a higher level of 5-hmC in their hippocampi when compared with the younger rats at 2 months of age (Fig.1E,F). Remarkably, hippocampal 5-mC content was negatively correlated with hippocampal 5-hmC and FA levels in rats of different ages (*R* = −0.793) (Fig.1G,H). Meanwhile, hippocampal 5-mC content was negatively correlated with hippocampal FA levels in rats of different ages (*R* = −0.900) (Fig.1H). These results suggest that aging-associated DNA demethylation is a source of hippocampal formaldehyde accumulation.

### Accumulated FA inactivates NE *in vitro* and *in vivo*

**Fig 2:**
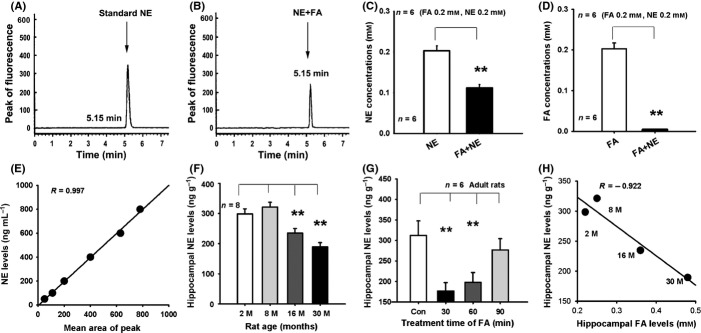
Accumulated FA inactivates NE *in vitro* and *in vivo*. (A, B) The peak of fluorescence of standard NE and of a solution of NE and FA. (C, D) The chemical reaction between NE and FA in PBS at 37 °C for 3 h (*n* = 6). (E) The standard curve of NE. (F) Hippocampal NE levels declined in aged rats (*n* = 8). (G) The metabolism of hippocampal NE after intrahippocampal injection of FA at 30, 60, and 90 min (*n* = 6). (H) Negative correlation between FA and NE level in hippocampus. **P <* *0.01.

To test the hypothesis that accumulated FA induces hippocampal NE deficiency by inactivating NE, we first examined whether there is a spontaneous chemical reaction between FA and NE *in vitro*. Using an ion-pair reversed-phase high-performance liquid chromatography with a fluorescence detector (RP-HPLC), we found that after FA was incubated with NE for 3 h, the NE concentration was reduced by half, and there was a complete reduction in FA levels in the solution mixture (Fig.1A–D). This result suggests that the chemical proportion of NE to FA is 1: 2 (Fig. S2). We then measured hippocampal NE concentrations in rats of different ages. Surprisingly, in the normal aging of rats from 2 to 30 months, there was a marked decrease in hippocampal NE concentrations (Fig.2E,F). To examine whether FA directly reduces hippocampal NE levels *in vivo*, we injected FA into the hippocampus of rats intrahippocampally and observed the metabolism of hippocampal NE. The results showed that injection of FA resulted in a marked decrease of hippocampal NE within 30 min, but a gradual recovery to baseline levels after one and a half hours (Fig.2G). Consistently, hippocampal NE concentrations were negatively correlated with hippocampal FA levels in rats of different ages (*R* = −0.922) (Fig.2H). These data indicate that excess FA reduces hippocampal NE levels.

### Depletion of NE impairs hippocampal LTP *in vivo*

**Fig 3:**
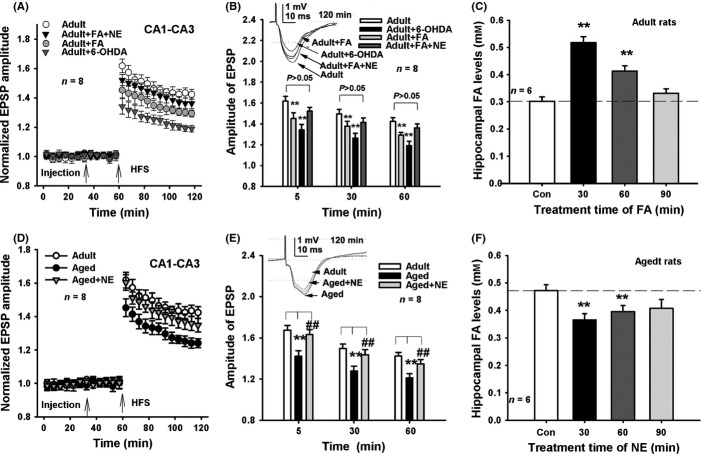
Depletion of hippocampal NE suppresses hippocampal LTP formation in healthy adult rats. (A, B) FA and 6-OHDA suppress LTP in the adult rats, but injection of NE rescues FA-induced LTP impairments (*n* = 8). (C) The metabolism of hippocampal FA after i.c.v. injection of FA at 30, 60, and 90 min (*n* = 6). (D, E) Rescue of LTP deficits in the aged rats after i.c.v. injection of NE (*n* = 8). (F) The metabolism of hippocampal FA after i.c.v. injection of NE at 30, 60, and 90 min (*n* = 6). *P <* *0.05; **P <* *0.01; ^##^*P *<* *0.01.

To mimic age-related NE deficiency, we injected 6-OHDA (a NE depletor) into healthy adult rats, and observed the effects on hippocampal LTP *in vivo*. LTP is regarded as a molecular basis of learning and memory. Our results showed that the maintenance of LTP was significantly different between the groups (*F*_3,28_ = 16.739, *P *<* *0.001) (Fig.3A). Intracerebroventricular (i.c.v.) injection of 6-OHDA prior to high- frequency stimulation (HFS) for half an hour in the adult rats, markedly suppressed hippocampal LTP formation compared with HFS treatment alone (Fig.3A,B). This result confirms that depletion of NE induces LTP impairment in adult rats. We then examined whether excess FA acts as well as 6-OHDA to suppress LTP and found that an i.c.v. injection of FA at 0.5 mm similarly impaired LTP in the adult rats. However, an i.c.v. injection of NE reversed FA-induced LTP impairments *in vivo* (Fig.3A,B). Moreover, we found that an FA injection induced a marked elevation in hippocampal FA levels after half an hour and a recovery to baseline levels after one and a half hours (Fig.3C). This last finding indicates that hippocampal FA approaches its maximum concentration half an hour after FA treatment.

If hippocampal NE deficiency in aged rats contributes to memory deficits, then a supplement of exogenous NE should rescue memory in these aged rats. To test this possibility, we injected NE into the aged rats and recorded LTP *in vivo*. The results showed that the maintenance of LTP was significantly different between the groups (*F*_2,21_ = 11.428, *P *<* *0.001). The adult rats at 3 months of age had a higher mean amplitude of LTP than that of the rats at 30 months of age, but an i.c.v. NE injection markedly reversed age-related LTP deficits in the aged rats (Fig.3D,E). This result is similar to that previously reported (Tully *et al*., 2007). More importantly, we found that an i.c.v. injection of NE can rapidly reduce hippocampal FA concentrations in aged rats after 30 min, but then recovered to baseline levels after one and a half hours (Fig.3F). This result again confirmed that FA can inactivate NE *in vivo*.

### Depletion of NE impairs spatial memory

**Fig 4:**
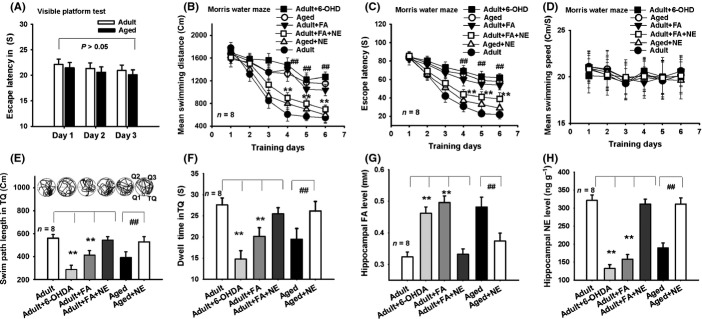
Depletion of hippocampal NE inhibits spatial memory formation in healthy adult rats. (A) No difference in visible platform test between the adult and aged rats. (B, C) Mean swimming distances and escape latency of the different drugs-treated rats in the Morris water maze. (D) No difference in swimming speed among the different drugs-treated rats. (E) A longer swimming distance shown by the FA- and NE-injected adult rats, and NE-injected aged rats as compared with the adult rats (*n* = 8). (F) Changes in time spent in the target quadrant (TQ, other quadrants were named as Q1, Q2, and Q3) for the different drugs-treated rats (*n* = 8). (G, H) Changes in hippocampal FA and NE concentrations for the different drugs-treated rats (*n* = 8). *P <* *0.05; **P <* *0.01; ^##^*P *<* *0.01.

As 6-OHDA depletes hippocampal NE content and leads to hippocampal LTP impairment, we further explore whether it can induce hippocampal FA accumulation and therefore mimic age-related memory deficits in healthy adult rats. In this study, we firstly found that there was no difference in visual capacity between the adult and aged rats in the visible platform test (Fig.4A). All rats showed a significant decrease in swimming distance and escape latency over the 6 days of acquisition trials in the Morris water maze (swimming distance: *F*_5,225_ = 118.317, *P *<* *0.001; escape latency: *F*_5,225_ = 67.493, *P *<* *0.001), which demonstrated that the rats learned the location of the platform in the acquisition trial. The day × treatment interaction was shown to be significant (swimming distance: *F*_25,225_ = 8.478, *P *<* *0.001; escape latency: *F*_25,225_ = 4.573, *P *<* *0.001), day (*F*_25,225_ = 17.329, *P *<* *0.001), and the main effect of treatment (different groups) was significant (swimming distance: *F*_5,42_ = 12.537, *P *<* *0.001; escape latency: *F*_5,42_ = 12.463, *P *<* *0.001), indicating that different treatment (FA, NE, 6-OHD, and FA with NE injection) influenced the spatial learning ability of adult and aged rats (Fig.4B,C).

Analyses of swimming distances within each day revealed a significant effect from the treatment on day 4 ((*F*_5,42_ = 8.762, *P *=* *0.001), day 5 ((*F*_5,42_ = 7.639, *P *=* *0.002), and day 6 ((*F*_5,42_ = 7.852, *P *=* *0.002). Post hoc analyses for day 4, 5, and 6 showed that the aged rats swam significantly further than the adult rats (*P *<* *0.01). There was no significant difference in swimming distance between the aged rat and adult rat treated with 6-OHD/FA, nor between the adult rat and aged rat treated with NE or the adult rat treated with a mixture of FA and NE. Analyses of escape latency within each day also showed a significant effect from the treatment on day 4 (*F*_5,42_ = 4.686, *P *=* *0.003), day 5 (*F*_5,42_ = 4.873, *P *=* *0.002), and day 6 (*F*_5,42_ = 5.104, *P *=* *0.001). Post hoc analyses for day 4, 5, and 6 showed that the aged rats had a longer escape latency than that of the adult rats (*P *<* *0.01). There was no significant difference in escape latency between the aged rat and adult rat treated with 6-OHD/FA, nor between the adult rat and aged rat treated with NE or the adult rat treated with a mixture of FA and NE (Fig.4B,C). Moreover, these differences in swimming distance and escape latency among the different groups did not result from swimming speed (Fig.4D).

On day 7 of the Morris water maze test, the 6-OHDA-injected or FA-injected adult rats showed a marked impairment in memory retrieval ability compared with the control adult rats (a shorter swimming distance and a less time spent in the target quadrant, *n* = 8; *P *<* *0.01; one-way ANOVAs). However, an intrahippocampal injection of NE not only reversed FA-induced memory deficits in the adult rats, but also rescued the age-related decline in the memory retrieval ability of the aged rats (a greater swimming distance and a longer time spent in the target quadrant, *n* = 8; *P *<* *0.01; one-way ANOVAs) (Fig.4E,F). This result is similar to that previously reported (Hatfield & McGaugh, 1999). These data indicate that age-related FA induces depletion of hippocampal NE levels and leads to memory decline in the aged rats.

We then observed the effects of an injection of 6-OHDA and FA on hippocampal FA and NE concentrations in the adult and aged rats. The results showed that injection of 6-OHDA or FA intrahippocampally into the adult rats induced a marked elevation in hippocampal FA levels compared to levels in the adult rats receiving no drug treatment. Injection of NE into the aged rats can reduce hippocampal FA concentrations as compared with those receiving no drug treatment (Fig.4G). Remarkably, injection of 6-OHDA or FA induced a marked decrease in hippocampal NE concentrations in the adult rats, and injection of NE elicited a rescue of hippocampal NE levels in the aged rats (Fig.4H). These findings indicate that FA-inactivated NE in the hippocampus contributes to age-related memory deficits.

### FA-induced NE deficiency impairs synaptic plasticity in SAMP8 mice

**Fig 5:**
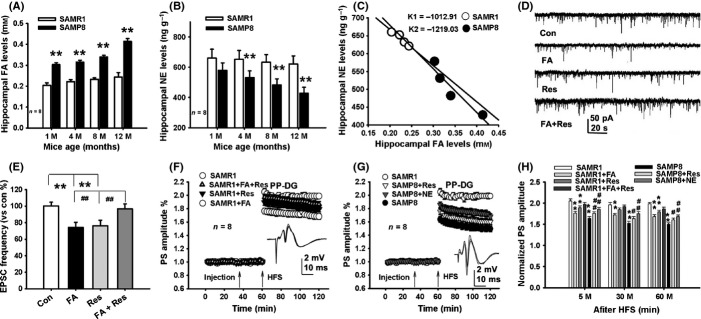
Accumulated hippocampal FA reduces NE levels and inhibits hippocampal synaptic plasticity of SAMP8 mice. (A, B) A marked increase in FA levels and a decrease in NE in the hippocampi of the SAMP8 mice compared with the SAMR1 mice. (C) A marked change in rake ratio of NE vs. FA levels in the SAMP8 mice than the SAMR1 mice. (D) The waves of EPSCs of hippocampal neurons after treatment with FA, Res, and FA with Res, respectively. (E) Effects of treatment with FA, Res, and FA with Res on the frequency of EPSCs (*n* = 10–15, recording neurons from 3 to 4 slices). (F–H) Effects of different drug treatments on hippocampal LTP in the SAMR1 and the SAMP8 mice (*n* = 8). *P <* *0.05; **P <* *0.01; ^##^*P *<* *0.01.

To further verify the hypothesis that FA-inactivated NE leads to age-related memory decline in aged rats, we used the senescence-accelerated prone 8 (SAMP8) mice to observe whether more severe FA-depleted NE and memory deficits occur in this senescence model compared with the senescence-resistant 1 (SAMR1) mice. The results showed that there was a marked increase in FA levels and a sharp decrease in NE concentrations in the hippocampi of the SAMP8 mice when compared with the SAMR1 mice (Fig.5A,B). Remarkably, the rank ratio of NE vs. FA in the hippocampi of the SAMP8 mice was sharper than that of the SAMR1 mice (Fig.5C). All these data suggest that the higher concentrations of endogenous FA accumulated in the hippocampus lead to the lower hippocampal NE levels during the process of accelerated aging.

As accumulated FA-inactivated hippocampal NE in aged rats (Fig.4A,B), scavenging excess FA may be a potential strategy for the treatment of age-related memory decline in the SAMP8 mice. First, we examined whether Res reverses FA-inhibited neuronal excitability in hippocampal slices from neonatal rats. By recording spontaneous excitatory postsynaptic currents (EPSCs), we found that application of FA at 0.5 mm or Res at 0.1 mm markedly suppressed the frequency of EPSCs, and that application of Res can reverse FA-inhibited effects on EPSCs (Fig.5D,E). By recording LTP in the perforant pathway-dentate gyrus (PP-DG) *in vivo*, the results showed that the maintenance of LTP was significantly different among the different drugs-treated SAMR1 mice (*F*_3,28_ = 16.482, *P *<* *0.001). FA injection markedly suppressed LTP formation, but, an i.c.v. injection of NE reversed FA-induced LTP impairments (Fig.5F). Similarly, the maintenance of LTP in the SAMP8 mice was significantly different among the different drugs-treated groups (*F*_3,28_ = 25.641, *P *<* *0.001). SAMP8 mice had a lower magnitude of LTP than the age-matched SAMR1 mice. However, an i.c.v injection of Res or NE into the hippocampus did reverse age-related LTP impairments in SAMP8 mice (Fig.5F–H). These data suggest that either scavenging FA from or supplying NE to the hippocampus rescues synaptic plasticity during aging.

### FA-induced NE deficiency leads to memory deficits in SAMP8 mice

**Fig 6:**
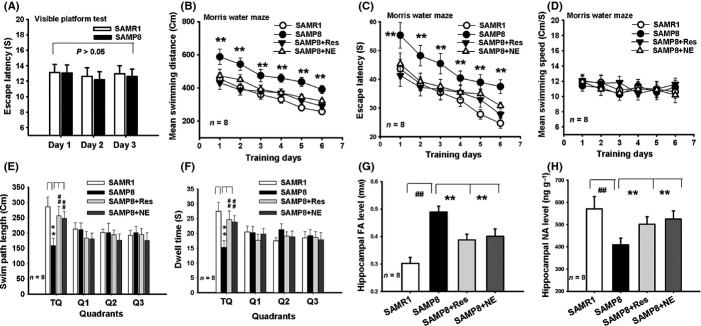
Scavenging FA or applying NE reverses age-related memory deficits in SAMP8 mice. (A) No difference in visible platform test between the SAMR1 and SAMP8 mice. (B, C) Mean swimming distances and escape latency of the different drugs-treated mice in the Morris water maze. (D) No difference in swimming speed among the different drugs-treated mice. (E) A longer swimming distance of the Res- and NE-injected SAMP8 mice as compared with the control SAMP8 mice (*n* = 8). (F) Changes in time spent in the target quadrant (TQ) for the different drugs-treated mice (*n* = 8). (G, H) Changes in hippocampal FA and NE concentrations for the different drugs-treated mice (*n* = 8). *P <* *0.05; **P <* *0.01; ^##^*P *<* *0.01.

In the Morris water maze experiments, we found that there was no difference in visual capacity between the adult and aged rats in the visible platform test (Fig.6A). The two-way repeated measures ANOVA for the swimming distance (*F*_5,75_ = 31.489, *P *<* *0.001) and the escape latency parameter revealed a main effect of day (*F*_5,75_ = 22.378, *P *<* *0.001), group (*F*_3,28_ = 16.488, *P *<* *0.001), and a day × group interaction (*F*_15,75_ = 7.324, *P *<* *0.001). Post hoc Dunnett tests showed a significant difference in swimming distance between the SAMP8 and the SAMR1 mice on acquisition day 1 (*F*_3,28_ = 11.297, *P *<* *0.001), day 2 (*F*_3,28_ = 10.318, *P *<* *0.001), day 3 (*F*_3,28_ = 12.247, *P *<* *0.001), day 4 (*F*_3,28_ = 12.875, *P *<* *0.001), day 5 (*F*_3,28_ = 9.796, *P *<* *0.001), and day 6 (*F*_3,28_ = 12.438, *P *<* *0.001). There was also a significant difference in escape latency between the SAMP8 and the SAMR1 mice on acquisition day 1 (*F*_3,28_ = 8.679, *P *<* *0.001), day 2 (*F*_3,28_ = 8.446, *P *<* *0.001), day 3 (*F*_3,28_ = 9.231, *P *<* *0.001), day 4 (*F*_3,28_ = 9.347, *P *<* *0.001), day 5 (*F*_3,28_ = 8.786, *P *<* *0.001), and day 6 (*F*_3,28_ = 9.214, *P *<* *0.001) (Fig.6B,C). Moreover, these differences in swimming distance and escape latency between the different groups were not a result of swimming speed (Fig.6D). These data indicate that the SAMP8 mice have lower spatial learning ability than the SAMR1 mice. Res or NE injection reversed age-related decline in learning ability of the SAMP8 mice.

On day 7 of the probe test, the SAMP8 mice demonstrated a poorer memory retrieval ability (a shorter swimming distance and less time spent in the target quadrant) compared with the SAMR1 mice. An injection of Res or NE rescued the memory performance of the SAMP8 mice (*n* = 8; *P *<* *0.01; one-way ANOVAs) (Fig.6E,F). Significantly, an injection of Res or NE induced a marked decrease in hippocampal FA, and an elevation in hippocampal NE concentrations in the SAMP8 mice (*n* = 8; *P *<* *0.01; one-way ANOVAs) (Fig.6G,H). These data indicate that it is FA-inactivated NE which leads to memory deficits in SAMP8 mice.

## Discussion

Aging-associated hippocampal NE deficiency has been found to be related to memory decline during aging and in patients with AD (Collier *et al*., 2004; Francis *et al*., 2012). Our recent studies have shown that hippocampal FA accumulation plays a critical role in cognitive decline in aged rats and in patients with AD (Tong *et al*., 2011, 2013a,b). In this study, our findings suggest that accumulated FA-inactivated NE in the hippocampus contributes to age-related memory decline.

### Age-related DNA demethylation leads to FA accumulation in the hippocampus

Recent high profile studies have found that maintaining the DNA methylation profile is necessary for maintaining long-term memory, because reduction (blockage) of global DNA methylation leads to long-term memory loss (Feng *et al*., 2010; Miller *et al*., 2010). Generally, aging is associated with a decrease in global DNA methylation levels. A decline in global DNA methylation levels (5-mC levels) was observed in cancer or proliferated cells (Lopatina *et al*., 2002; Ehrlich, 2009), and in both aged mice and rats associated with memory decline (Liu *et al*., 2009; Oliveira *et al*., 2012), in particular, of autopsied brain samples taken from patients with AD suffering from cognitive disorder (Mastroeni *et al*., 2011; Chouliaras *et al*., 2013). Surprisingly, there was an elevation in FA concentration in the proliferated cancer cells (Tong *et al*., 2010), in aged brains (Tong *et al*., 2013b), and in the autopsied brain samples from patients with AD (Tong *et al*., 2011). Recent studies have established the relationship between age-related DNA demethylation (i.e., a decrease in 5-mC in DNA) and FA accumulation. DNA demethylation (i.e., a decrease in 5-mC level) leads to formaldehyde generation via different pathways: one pathway is that TeT1-mediated DNA demethylation can transform 5-mC to 5-hmC, which then leads to FA generation (Wu & Zhang, 2010; Guo *et al*., 2011) (Fig. S3A); another pathway of FA generation is thought to be mediated by demethylases (Patra *et al*., 2008) (Fig. S3B). Our previous study also found that external stimulation-mediated DNA demethylation induced a Ca^2+^-dependent FA generation (Tong *et al*., 2013a). In this study, 5-mC concentrations were negatively correlated with both 5-hmC and FA levels in the hippocampi of rats of different ages, supporting the notion that age-related DNA demethylation leads to FA accumulation. However, the exact mechanism of aging-induced FA generation still needs further elucidation.

### Accumulated FA induces hippocampal NE deficiency

The interesting finding in this study is that aging-associated FA induces hippocampal NE deficiency by inactivating NE. We found that NE was rapidly inactivated by FA in a phosphate buffer solution at 37 °C, this result being similar to findings from a previous study (Tani, 1981). However, the exact mechanism of the chemical reaction between NE and FA is unclear. Using a Fluo-HPLC to detect the FA concentration and a RP-HPLC to measure the NE levels, we found that the proportion of chemical reaction between NE and FA was 1: 2 (Fig. S4A). Another earlier study has given a reasonable theoretical explanation (Jonsson, 1969; Einarsson *et al*., 1975) (Fig. S4B), and this reaction has been verified by synchronous fluorimetry to detect NE concentration (Guo *et al*., 2005). Remarkably, FA, a small molecule (MW = 30), can efficiently traverse cell membranes as well as the blood–brain barrier (Tulpule & Dringen, 2012). However, the neurotransmitter, NE, is transported by synaptic vesicles. These factors provide a possible explanation for the high concentrations of FA, and the low levels of NE that can be detected in the hippocampus. This in turn suggests that FA rapidly inactivates NE in the synaptic cleft. However, the exact mechanism needs to be further investigated. We also found that an intrahippocampal injection of NE markedly reduced the hippocampal FA concentration and, conversely, that an intrahippocampal injection of FA decreased hippocampal NE levels. Moreover, there was an inverse relationship between FA and NE levels in the hippocampi of healthy rats at different ages. Collectively, accumulated FA can induce NE deficiency by inactivating NE *in vitro* and *in vivo*.

### FA inactivates NE resulting in memory disorder in the elderly and in early-stage AD

Another important finding is that FA-inactivated NE contributes to age-related memory deficits. In this study, age-related LTP and memory deficits associated with both hippocampal FA accumulation and NE deficiency were observed in the aged rats and SAMP8 mice. Previous studies have shown that depletion of hippocampal NE, by injection of 6-OHDA (a NE depletor) or DSP-4 (a norepinephrinergic neurotoxin), impairs LTP and memory in adult rats (Stanton & Sarvey, 1985; Lapiz *et al*., 2001). In the present study, injection of FA as well as 6-OHDA into the adult rats markedly reduced hippocampal NE content and mimicked age-related deficits in LTP and memory. Therefore, inactivation of hippocampal NE by accumulated FA is a reasonable explanation for hippocampal NE deficiency-related memory decline in aged rats. On the other hand, we further provided evidence that supplementing NE not only reduces hippocampal FA levels but also reverses age-related deficits in LTP and memory. Similarly, other studies have shown that an injection of NE and transplantation of norepinephrine neurons into aged rats can restore the magnitude of LTP and memory performance in these rats (Collier *et al*., 1988; Almaguer-Melian *et al*., 2005). More importantly, in this study, the accelerated senescence model SAMP8 mice showed a more severe decrease in NE content and a rapid increase in FA levels in the hippocampus. These results are similar to results previously reported (Huang *et al*., 2010; Tong *et al*., 2011). However, scavenging FA by intrahippocampal injection of exogenous NE or Res (a natural FA scavenger) can reduce hippocampal FA and partially restore hippocampal NE levels, reversing deficits in LTP and memory in the SAMP8 mice. Clinical investigation has found that endogenous FA is initially elevated in patients with AD with mild cognitive impairment (MCI) (Tong *et al*., 2011). These data along with our results suggest that FA-inactivated NE is an early etiological factor for cognitive disorder in the elderly and patients with AD.

## Experimental procedures

### Animals

All protocols involving the use of animals were conducted in accordance with the Biological Research Ethics Committee, Capital Medical University, China. Male Sprague–Dawley rats at 2, 8, 16, and 30 months of age were obtained from the Experimental Animal Center of Peking University, China, and the SAMR1/SAMP8 male adult mice at 1, 4, 8, and 12 months of age were provided by the Weitong Lihua Animal Center, China. All the animals were maintained in cages at room temperature (25 °C) under an alternating 12 h light/dark cycle (lights on at 7:00), with *ad libitum* access to food and water.

### Reagents

All reagents were from Sigma unless otherwise indicated.

### Intracerebroventricular injection in the SD rats

The SD rats were injected with drugs, such as FA (0.5 mm, 5 μL was injected for 5 min), NE (10 μm, 5 μL, over a period of 5 min), FA with NE (5 μL, a solution mixture of 0.5 mm FA and 0.25 mm NE), or 6-OHDA (8 μg, 5 μL, over a period of 5 min), for recording hippocampal LTP *in vivo* or for detecting hippocampal NE and FA levels. Cannulae placements in brains of rats: 0.8 mm anterior/posterior to bregma, 1.2 mm medial/lateral to the midsagittal suture, and 2.0 mm dorsal/ventral from the brain surface (Tong *et al*., 2013a).

### *In vivo* LTP recording in rats

The 3-month-old young adult or 30-month-old aged male Sprague–Dawley (SD) rats were surgically prepared for hippocampal LTP recordings *in vivo* as described previously (Tong *et al*., 2013b). Briefly, the rats were anesthetized with an intraperitoneal (i.p.) injection of 1.5 g kg^−1^ urethane (ethyl carbamate) in a stereotaxic frame. Rats were positioned in the stratum radiatum of area CA1 (3.4 mm posterior to bregma and 2.5 mm lateral to the midline); a bipolar concentric stimulating electrode was placed in the Schaffer collateral-commissural pathway of CA3 (4.2 mm posterior to bregma and 3.8 mm lateral to the midline) distal to the recording electrode (Fig. S1A,B). Bipolar tungsten stimulating electrodes and monopolar tungsten recording electrodes were obtained from A-M SYSTEMS (Carlsborg, WA, USA). LTP was induced using a high-frequency stimulation (HFS) protocol consisting of 20 pulses at 100 Hz. A >50% increase in fEPSP amplitude (Fig. S1C), from the baseline, was considered to be a significant LTP.

### Bilateral hippocampal injection in the SD rats

SD rats were anesthetized via i.p. injection of a ketamine (30 mg kg^−1^) and xylazine (2.5 mg kg^−1^) solution with supplemental injections given as needed. Under aseptic conditions, a stereotaxic instrument was used to implant 23 gauge guide cannulae bilaterally into the dorsal hippocampus. Cannulae placements in the dorsal hippocampus, 3.6 mm anterior/posterior to bregma, 2.5 mm medial/lateral to the midsagittal suture, and 2.5 mm dorsal/ventral from the brain surface. The site of drug injection was identified by paraffin section (Fig. S1D) (Tong *et al*., 2013a). These rats were injected intrahippocampally with normal saline, FA (0.5 mm, 2 μL) was injected for 5 min, NE (10 μm, 2 μL), or 6-OHDA (8 μg, 2 μL) half an hour before the daily behavioral experiments in a Morris water maze or for the detection of metabolism of NE and FA in the hippocampus.

### Morris water maze behavioral test for rats

Spatial memory behaviors were assessed by the Morris water maze test, as described previously (Morris, 1984; Tong *et al*., 2013a). All rats were trained to mount a hidden/submerged (1.5 cm below the surface for rats) escape platform in a restricted region of the pool (Pool diameter: 1.8 m for rats). Spatial memory was assessed by recording the latency time for the animal to escape from the water onto the submerged escape platform as a function of the number of learning trials during the learning phase. After 6 days of learning, the rats were subjected to a 90-s probe trial wherein the escape platform was removed on day 7. The water maze activity was monitored with the Instrument Poly-Track video-tracking system (San Diego Instruments, California, USA).

### Rat hippocampus slice preparation

Neonatal rats were anesthetized in a CO_2_ chamber or anesthetized deeply with methoxyflurane before rapid decapitation as described previously (Tan *et al*., 2013).

### Recording spontaneous discharge *in vitro*

Patch pipettes (3–5 MΩ, after filling with intrapipette solution) were pulled from borosilicate glass using a horizontal puller (P-97, Sutter Instrument Company, Novato, USA). All experiments were carried out at room temperature (25 °C). After achieving the whole-cell configuration, leakage and capacitative currents were subtracted online using a P/4 procedure. Series resistance was in the range of 10–30 MΩ and compensated by 60–80% (20 ms lag time) during voltage-clamp recordings. sEPSCs were recorded at a holding potential of -70 mV (Tan *et al*., 2013), using the standard extracellular solution, and pipettes were filled with a solution containing (in mm) 144 KCl, 3 EGTA, 5 NaCl, 1 MgCl_2_, 10 HEPES, and 2 Na_2_ATP, pH was adjusted to 7.2 with KOH.

### Detecting FA by Fluo-HPLC

Half of all the hippocampal samples of mice and rats after electrical stimulation or spatial memory behavior tests were collected and immediately placed on ice before storing at −70 °C. After centrifuging (6000g, 4 °C, 10 min), supernatant fractions of brain homogenates (weight of brain tissue: ultrapure water = 1:4) were analyzed by high-performance liquid chromatography with fluorescence detection (Fluo-HPLC) as described previously (Luo *et al*., 2001; Tong *et al*., 2011).

### Detecting NE by RP-HPLC

The other half of the hippocampal samples were used for measuring NE concentration by an ion-pair reversed-phase high-performance liquid chromatography with a fluorescence detector (RP-HPLC) as previously described (Yamaguchi *et al*., 1998). Briefly, the column chamber temperature was set at 28 °C and the sample volume was set at 10 μL. The excitation and emission wavelengths of the fluorescence detector were set at 280 nm and 340 nm, respectively. The mobile phase was KH_2_P_3_O_4_: acetonitrile (98: 2, v/v; pH = 4.0) with a flow rate of 0.8 mL min^−1^. The HPLC column was a PH-BDS C18 (5 μm, 250 mm × 4.6 mm). The peak area was used for quantitative calculation.

### Intracerebroventricular injection in mice

The SAMR1 and SAMP8 adult male mice at 12 months of age were given an i.c.v. injection of drugs such as normal saline, NE (10 μm, 2 μL was injected for 4 min), or Res (100 μm, 2 μL), for recording hippocampal LTP *in vivo* or for detecting hippocampal NE and FA levels. Cannulae placements in brains of mice: 0.5 mm anterior/posterior to bregma, 1.0 mm medial/lateral to the midsagittal suture, and 2.0 mm dorsal/ventral from the brain surface.

### *In vivo* LTP recording in mice

Electrophysiological recordings from perforant pathway to the dentate gyrus (PP-DG) in the hippocampi of the SAMR1 and SAMP8 adult male mice at 12 months of age were performed according to previous reports (Bliss & Errington, 1984; Huang *et al*., 2008). Briefly, held under by 20% urethane anesthesia (1.5 g kg^−1^, i.p.), the rats were placed in a stereotaxic frame. Population spikes (PS) were then evoked using a tungsten bipolar stimulating electrode that was positioned in the perforant path (PP) (anterior posterior, 3.8 mm; lateral, 3.0 mm; horizontal, 1.5 mm from the bregma). These spikes were recorded using a 2 mol L^−1^ sodium chloride-filled glass pipette that was positioned in the granular cell body region of the dentate gyrus (DG) (anterior posterior, 2.0 mm; lateral, 1.4 mm; horizontal, 1.5 from the bregma) using an Axoclamp-2B amplifier (Axon Instruments, Foster City, California, USA) (Fig. S2A–B). Baseline responses were set to 50% of maximal response and recorded for 30 min. LTP was induced by tetanic stimulation (the parameter: 3 trains of 8 pulses (400 Hz, 400 μs) at 10 s intervals) using an Electronic Stimulator (MSE-3, Nihon Kohden, Japan). Evoked responses recorded before (30 min) and after (1 h) LTP induction were used for the analysis of population spike amplitude (PS) (Fig. S2C). The peak amplitude of the PS was the mean percentage change in 5 min. Data were analyzed using Clampfit 10 (Molecular Devices, Sunnyvale, California, USA) and were presented as mean ± SEM. Responses were normalized to baseline, and data were analyzed using ANOVA.

### Bilateral hippocampal injection in mice

Thirty-two mice were anesthetized with isoflurane and placed in aseptic conditions. Implanted cannulae were cemented on the skull. Drugs in 2 μL volume were injected bilaterally into the dentate gyrus region of the hippocampus using the following coordinates: 2.0 mm anterior/posterior, 1.5 mm medial/lateral, and 1.7 mm dorsal/ventral from the bregma. The drugs were injected at 0.5 μL min^−1^ over a period of 4 min using a Hamilton 5-μL syringe and a 27 G needle. The site of drug injection was identified by paraffin section (Fig. S2D). The drug-injected rats were injected intrahippocampally half an hour before the daily behavioral experiments in a Morris water maze or for the projects for detecting metabolism of NE and FA in the hippocampus.

### Morris water maze behavioral test for mice

Spatial memory behaviors of the SAMR1 and SAMP8 male mice at 12 months of age with or without drug treatment were assessed using the Morris water maze test, as described previously (Morris, 1984; Tong *et al*., 2011).

### Quantification of global 5-mC and 5-hmC levels

Nuclei were extracted from rat hippocampi using an EpiQuik™ nuclear extraction kit (Epigentek, Brooklyn, New York, USA). The global 5-mC and 5-hmC in DNA were quantified using the MethylFlash Methylated DNA Quantification Kit (Cat #P-1034, Epigentek) and the MethylFlash Hydroxymethylated DNA Quantification Kit (Cat #P-1036, Epigentek) according to the manufacturer’s instructions.

### Statistical analysis

The changes in response amplitudes of LTP were analyzed using mixed design ANOVAs. In the Morris water maze experiment, measures of performance during acquisition trials (i.e., escape latency) were averaged within each day for each animal. To determine the difference between each day, data were analyzed using two-way repeated measures ANOVAs, with day as the within-subjects factor, and different treatment as the between-subjects factor. The difference between the different treatment groups within each day was analyzed with a one-way ANOVA test, and Fisher’s LSD was used for post hoc comparisons. SPSS 16.0 (SPSS Inc., Chicago, IL, USA) was used for all analyses. For other experiments, statistical significance was determined by means of the Student’s *t*-test (for independent or dependent samples, as appropriate) with *P *<* *0.05 (two-tailed) considered. Data are reported as mean ± standard errors.

## Funding information

This research was supported by grants from the fund of the Beijing Institute for Brain Disorders (0000040103), the Scientific Research Common Program of Beijing Municipal Commission of Education (KM201510025014), the Natural Scientific Foundation of CCMU (2015ZR31), and the Natural Scientific Foundation of China (NSFC 31171080, 51136002).

## Conflict of interest

None declared.

## Supporting Information

**Fig. S1**Positioning of two-electrodes and trace of intrahippocampal injection in SD rats. **Fig. S2** Positioning of two-electrodes and trace of intrahippocampal injection in mice. **Fig. S3** Two possible pathways of DNA demthylation leading to endogenous formaldehyde generation. **Fig. S4** The chemical reaction between NE and FA.
