# Supplementary material for: Aging-associated formaldehyde-induced norepinephrine deficiency contributes to age-related memory decline
Source: Aging Cell. 2015 Apr 11;14(4):659–68. doi: 10.1111/acel.12345 (PMC4531079; doi:10.1111/acel.12345)
Supplement: Supplementary file 1 [file acel0014-0659-sd1.pdf]

Supplementary Figure 1

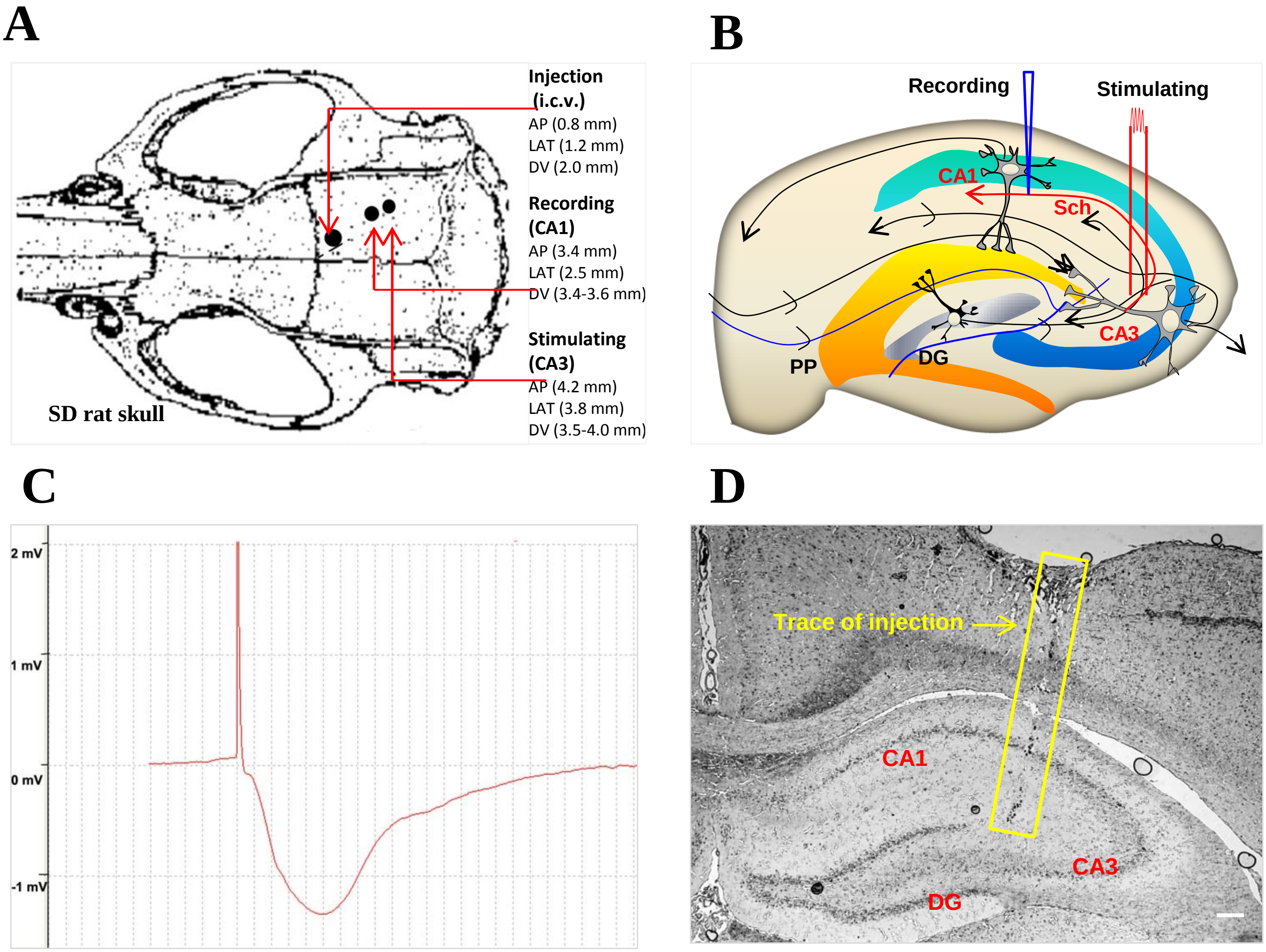

Figure 1. Positioning of two-electrodes and trace of intrahippocampal injection in SD rats.

(**A, B**) Scheme depicting the accurate positions of the cannulae in the cerebroventricular, the stimulating electrode in the Schaffer collateral-commissural pathway of CA3, and the recording electrode in the stratum radiatum of area CA1 in skulls of adult SD rats. (**C**) The standard wave of fEPSP recorded in the CA1-CA3 pathway of hippocampi of SD rats. (**D**) Trace of intrahippocampal injection of SD rats.

Supplementary Figure 2

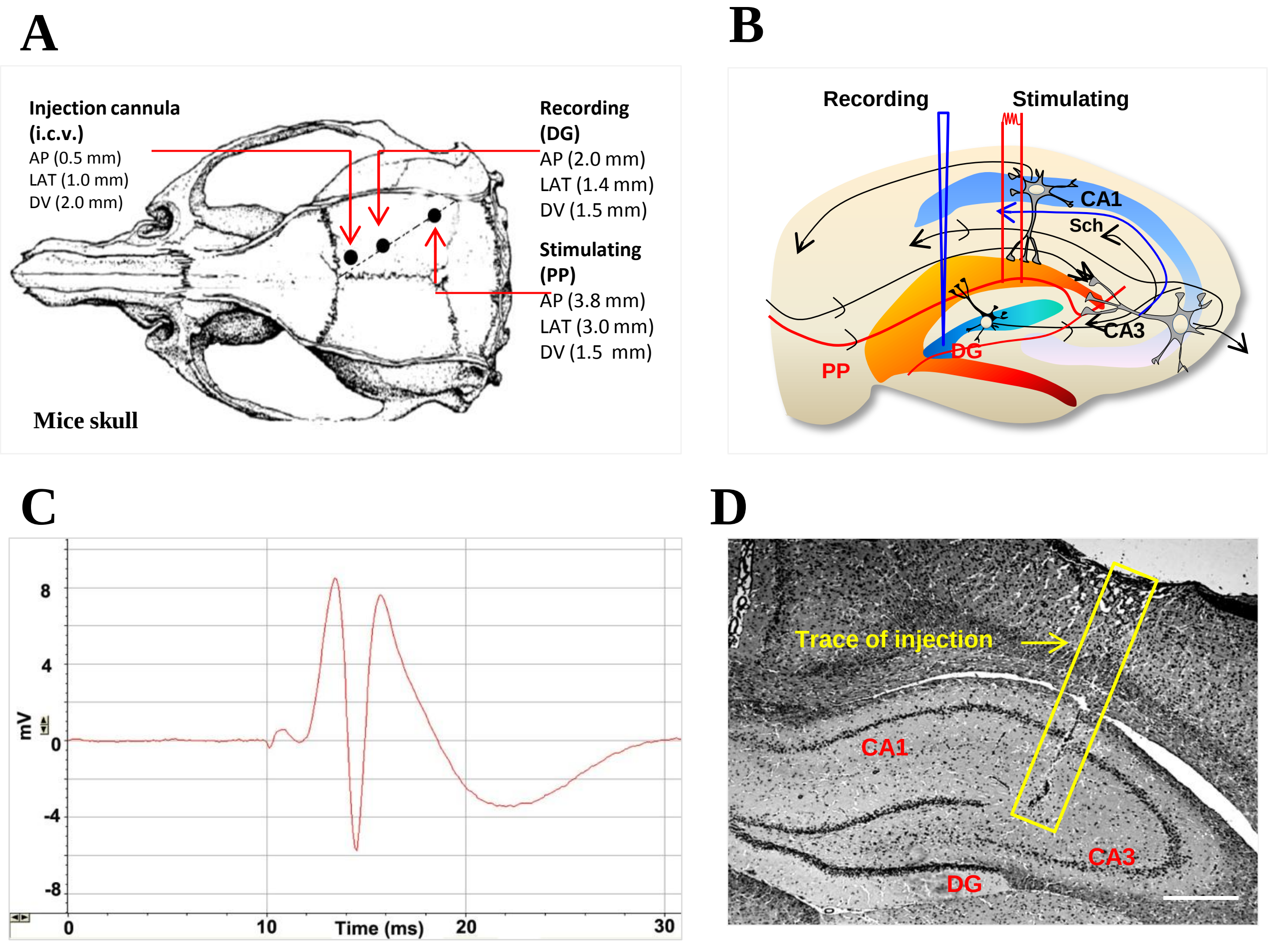

Figure 2. Positioning of two-electrodes and trace of intrahippocampal injection in mice.

(**A**, **B**) Scheme depicting he accurate positions of the injection cannulae in the cerebroventricular, the stimulating electrode in the perforant path (PP), and the recording electrode in the dentate gyrus (DG) in skulls of mice. (**C**) The standard wave of population spike (PS) recorded in the PP-DG pathway of the hippocampi of mice. (**D**) Trace of intrahippocampal injection in the hippocampi of mice.

Supplementary Figure 3

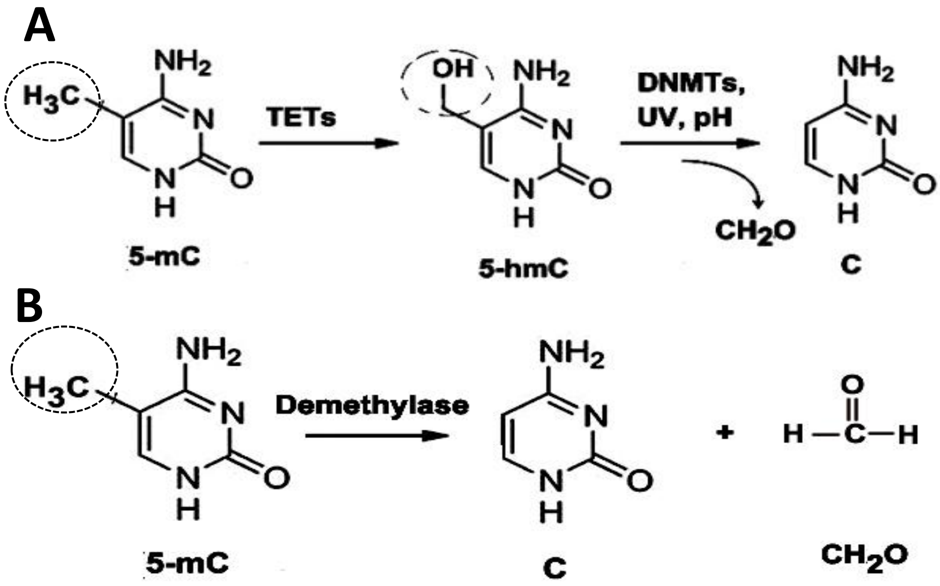

Figure 3. Two possible pathways of DNA demthylation leading to endogenous formaldehyde generation.

(A) TeT1-mediated DNA demethylation can transfer 5-mC to 5-hmC, and then leads to FA generation (Wu SC 2010; Guo JU2011).

(B) DNA demethylation induces FA generation mediated by demethylases (Patra *et al.* 2008).

Abbreviation: cytosine, C; formaldehyde, FA or CH_2O; 5-methylcytosine, 5-mC; 5-hydroxymethylcytosine, 5-hmC; ten-eleven translocation enzymes, TeTs; DNA methyltransferases, DNMTs; ultraviolet, UV

Supplementary Figure 4

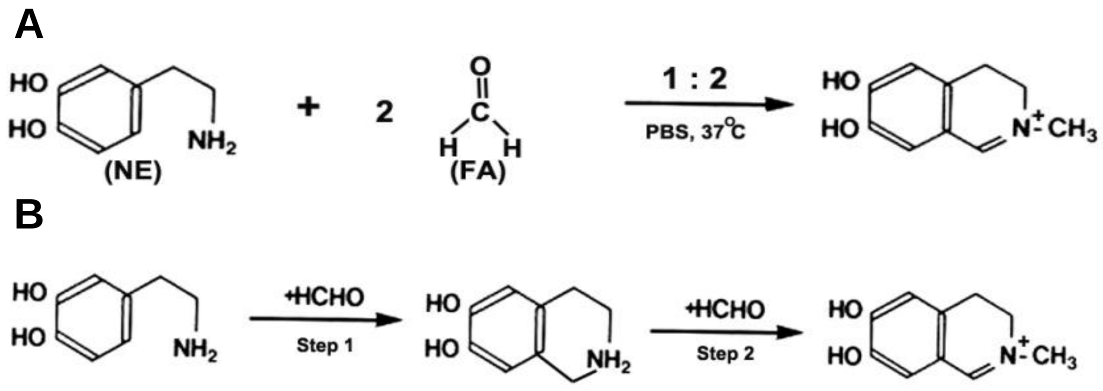

Figure 4. The chemical reaction between NE and FA.

(A) The proportion of chemical reaction between NE and FA was 1: 2.

(B) In the first step, a non-fluorescent substance tetrahydroisoquinoline (II) is formed, after NE and FA are incubated; in a second step, two types of dehydrogenated isoquinolines (III) are formed (Jonsson 1969; Einarsson *et al.* 1975)

Abbreviation: formaldehyde, FA; norepinephrine, NE
